# Curriculum Innovations: “Eye”-ing Enhanced Educational Methods for Neurology Trainees: Pilot of an Online, Case-based Neuro-ophthalmology Interactive Workshop

**DOI:** 10.1212/NE9.0000000000200052

**Published:** 2023-03-07

**Authors:** Emma M. Loebel, Laura K. Stein, Michael Fara, Samira Farouk, Nisha Chadha

**Affiliations:** From the Icahn School of Medicine at Mount Sinai, New York City.

## Abstract

**Introduction and Problem Statement:**

Common and potentially life-threatening neurologic conditions often present with neuro-ophthalmologic manifestations. Given the growing shortage of neurologists, and specifically neuro-ophthalmologists, it is important that students who will be at the front lines of these complaints are comfortable assessing such patients. We developed a neuro-ophthalmology learning intervention composed of an interactive workshop that discussed novel, online case-based modules. We assessed (1) the subjective and objective improvement in the understanding of neuro-ophthalmologic manifestations of common neurologic conditions and (2) satisfaction with the educational tool.

**Objectives:**

The objectives of this study were to identify and describe common neuro-ophthalmologic manifestations of neurologic conditions, to explain the differential diagnosis, diagnostic workup, and evidence-based treatment of common neuro-ophthalmologic conditions, and to use interactive, case-based discussion to foster an enjoyable, student-focused e-learning environment via the 20/20 SIM platform.

**Methods and Curriculum Description:**

Our study team, composed of ophthalmology and neurology faculty, developed cases for 5 common and high-stake neurologic conditions with neuro-ophthalmologic manifestations and published them on 2020SIM.com. The cases served as the basis of our educational intervention, a 1-hour virtual interactive workshop for neurology clerkship students. Students completed optional, anonymous pretests and posttests and an exit survey to assess subjective and objective neuro-ophthalmology knowledge improvement and satisfaction with the educational tool.

**Results and Assessment Data:**

A total of 145 students participated; 86% (n = 125), 70% (n = 102), and 61% (n = 88) completed at least part of the pretest, posttest, and exit survey, respectively. The mean knowledge score increased from 7.5 to 8.5/10, *p* = 0.00014. Students reported a subjective increase in knowledge of neuro-ophthalmology (70%, n = 62) and wished to see a similar learning tool for other specialties (92%, n = 81). More than half (64%, n = 56) enjoyed the workshop, approximately three-quarters (73%, n = 64) preferred the interactive session to traditional didactics, and almost all (92%, n = 81) students recommended the learning sessions in the future.

**Discussion and Lessons Learned:**

Medical students experienced subjective and objective improvement in their understanding of neuro-ophthalmologic manifestations of common neurologic conditions. In addition, they rated the intervention favorably in relation to traditional didactics and recommended a similar platform in other specialties. The integration of interactive online learning tools, such as the SIM platform, into curricular workshops may offer a favorable and effective strategy to increase exposure to topics with less curricular time.

## Introduction and Problem Statement

A number of common and life-threatening neurologic conditions may initially manifest with ophthalmologic signs and symptoms, and timely detection of these diseases can help prevent devastating consequences.^1^ In light of the growing shortage of neurologists,^2^ and specifically neuro-ophthalmologists,^3^ it is imperative to introduce students who will be at the front lines of these complaints to high-yield neuro-ophthalmologic conditions.

Despite the call from the American Academy of Neurology that neurology students should be competent in recognizing, evaluating, and managing certain urgent ophthalmologic conditions such as acute vision loss that may be due to brain pathology,^4^ exposure to neuro-ophthalmology is insufficient from the start. Only 84% of medical schools require a neurology rotation,^5^ and the minority (16%) require clinical rotations in ophthalmology.^6^ The United States urgently needs more neuro-ophthalmologists^7^ and based on the dearth of ophthalmology-related requirements in medical school,^6^ it is not clear that most trainees gain exposure to this important field. Even neurology residents have cited “lack of exposure” as a leading reason to not pursue the subspeciality.^3^ Our institution is part of most medical schools that do not require an ophthalmology clerkship,^6^ and neuro-ophthalmology was not part of our neurology clerkship curriculum previously.

In addressing this education gap, we acknowledged “neurophobia,”^8^ or the fear of neurology. Contributing to this fear is “poor teaching” that does not adequately link basic sciences to real-world applications.^9^ The literature suggests that learners gain and retain knowledge to a greater degree when taught in interactive formats,^10^ and medical students particularly may learn better when taught through case-based formats.^11^ Early experience with case-based learning (CBL) in ophthalmology has demonstrated higher enjoyment and ability to apply and retain knowledge.^12^ Incorporating CBL in neurology education during medical school may help reduce neurophobia^13,14^ and ultimately improve the recognition of common neurologic disorders by the physicians at the front lines.

Virtual learning instruments, often referred to as “e-learning,” have risen in popularity in medical education as teaching becomes more tech enabled, student driven, and student centric. Such tools span an increasingly diverse set of formats (e.g., cases, podcasts, discussion boards, and question banks) and have shown to successfully help bridge the gap from preclerkship textbook learning to real-world practice.^11,15^ In keeping with Adult Learning Theory,^16^ online platforms with hyperlinks can help learners review basic concepts and redirect to additional sources to meet one's individual educational needs. Through the implementation of free open-access medical education (FOAMed) devices using online platforms, anyone, at any time, can have access to high-quality peer-reviewed content.

We sought to bring case-based e-learning to clerkship year medical students to enhance and standardize exposure to neuro-ophthalmologic manifestations of common neurologic conditions during the neurology clerkship. We used 20/20 SIM (2020SIM.com), a case-based ophthalmology learning tool and FOAMed website, modeled off the SIM platform.^17^ Our educational intervention consisted of a 1-hour virtual interactive workshop for neurology clerkship students, highlighting common neurologic conditions with neuro-ophthalmologic manifestations and guided by online modules. We aimed to assess (1) the subjective and objective improvement in understanding of the neuro-ophthalmologic manifestations of common neurologic conditions during the neurology clerkship and (2) satisfaction of the educational tool. We hypothesized that participants would modestly improve their objective knowledge of neuro-ophthalmologic manifestations of common neurologic conditions as well as show satisfaction with our FOAMed tool and integrated workshop.

## Objectives

The objectives of this study were to identify and describe common neuro-ophthalmologic manifestations of neurologic conditions, explain the differential diagnosis, diagnostic workup, and evidence-based treatment of common neuro-ophthalmologic conditions, and use interactive, case-based discussion to foster an enjoyable, student-focused e-learning environment via the 20/20 SIM platform.

## Methods and Curriculum Description

### Development of Study Team

Our study team consisted of 1 ophthalmology (N.C.) and 2 neurology faculty members (L.K.S. and M.F.) and 1 third-year medical student (E.M.L.) at the Icahn School of Medicine at Mount Sinai (ISMMS). One member (N.C.) cofounded 20/20 SIM (2020SIM.com), which was initially developed as an online CBL tool for general ophthalmology. All study members had experience working in medical student education.

### General Needs Assessment

Literature search was performed to identify educational needs in medical student curricula within the field of neuro-ophthalmology. We reviewed neurology^4,18-20^ and ophthalmology^6^ educational guidelines and reference materials to identify common learning objectives and then developed a list of suggested clinical competencies for medical students that relate to neuro-ophthalmology. In addition, we reviewed outlines and sample practice questions from national testing platforms such as the National Board of Medical Examiners and United States Medical Licensing Examination.^21,22^ Finally, we explored the neurology and ophthalmology content in question banks including UWorld^23^ and AMBOSS.^24^

### Selection of Cases

We developed 5 cases to highlight common neurologic conditions with neuro-ophthalmologic manifestations, informed by our literature review of ophthalmology and neurology educational guidelines, medical student question banks, and expert faculty opinion.^4,6^ The cases included (1) central retinal artery occlusion, (2) idiopathic intracranial hypertension, (3) optic neuritis, (4) cranial nerve III palsy, and (5) superior quadrantanopia. We aimed to choose cases that would give students transferable skills for other neurologic and neuro-ophthalmologic conditions. The cases were labeled by number so students would not know the diagnosis until the end of the module.

### Development and Publication of Cases

**Figure 1:**
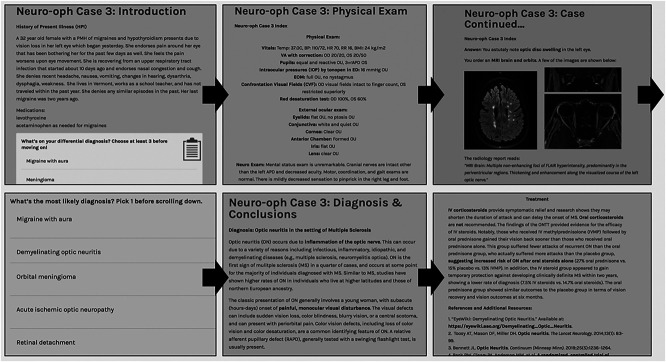
The Interactive Stepwise Case Flow Cases opened with a history of present illness, followed by physical examination findings, imaging and/or laboratory values, and concluded with a discussion on diagnosis and treatment including hyperlinks to further material and references. Optional questions are dispersed throughout the steps of the case.

Each case module aimed to mimic real-world clinical practice and was written in an interactive format with immediate feedback. Modules opened with a history of present illness (HPI), followed by physical examination, imaging studies, and laboratory values (Figure 1). Multiple choice questions were interspersed throughout each case, encouraging participants to choose the next best step before progressing to additional information. All questions were optional, so students could still move forward without answering. Students were asked to form a differential diagnosis after the HPI and then were given further opportunities to refine the differential as more information was provided. Each case module ended with a diagnosis and conclusion, briefly highlighting important teaching points and seminal studies on the condition. The final page included hyperlinks to related additional learning content (Figure 1). All the answers to the case questions were provided by the end of the module. These web-based modules were constructed with the intention of serving as an independent study resource that could be adapted for use in an interactive workshop format.

### Distribution of Cases Through the SIM Platform

The SIM platform, a mobile-optimized FOAMed website, was initially developed and cofounded by 1 coauthor (S.F.) for nephrology medical student education in the form of NephSIM (NephSIM.com). Through mass online distribution, thousands of users used the modules. Nearly all were fond of the platform, with 96% (n = 73) both enjoying the website and citing its ease of use and (99%, n = 75) indicating a desire to revisit in the future.^17^ 20/20 SIM was the first adaptation in the SIM series, modeled off of NephSIM, and initially launched with cases of ophthalmology for primary care in 2020. The neuro-ophthalmology cases were added as a novel series on 2020SIM.com, using Wordpress, a web publishing software used across all the SIM series sites.^17^ The study team created a distinct neuro-ophthalmology section to encourage focused learning in neuro-ophthalmology. We used this FOAMed format so that the cases and educational value could be extended to learners worldwide without geographic or financial constraints.

### Implementation of the Web-based Modules in a Learning Session

At the ISMMS, every third-year student rotates through a 4-week required neurology clerkship. From July 2020 to June 2021, neurology clerkship students participated in a 1-hour interactive workshop during the first week of the clerkship. The session centered on the learners and served as a standardized introduction to the 20/20 SIM website. Each session was attended by at least 1 faculty member of the study team and walked through 2–3 modules on 2020SIM.com of the participants' choice.

Each session opened with a short verbal introduction (2–3 minutes) explaining why learning neuro-ophthalmological cases is important to all medical trainees. The session facilitator then shared the screen of the 2020SIM.com home webpage and had any student volunteer choose a case number to start with at random. The facilitator asked for students to read the case stems and subsequent questions out loud to the group. The facilitator asked students to comment on answer choices throughout the case, including reasoning for or against a given option and the likelihood of a specific diagnosis. Once all the answer options were discussed, the facilitator would lead the group to the conclusion section for review of essential concepts, opportunities for additional self-directed learning via included hyperlinks, and discussion of additional questions, as needed. Each case required approximately 15–25 minutes for completion, allowing for the discussion of 2–3 cases in the 1-hour time slot. During the session, students were encouraged to visit 2020SIM.com on their own time for additional learning.

Case hits were tracked by WordPress and defined by the number of visits to the introduction page of each case. Case completion rates were calculated as the total number of visits to the introduction page divided by those to the conclusion page.

### Evaluation of Learner Knowledge Gain and Satisfaction

Students completed the optional, anonymous 10-question knowledge pretest prior to discussion of cases. During the fourth week of the clerkship, students completed an identical 10-question knowledge posttest. The knowledge questions covered clinical case scenarios similar to those on 20/20 SIM platform, including central retinal artery occlusion, idiopathic intracranial hypertension, cranial nerve III palsy, and bitemporal hemianopia secondary to pituitary adenoma.

In addition, in the fourth week of the clerkship, students had the option to complete an exit survey to assess subjective gains and satisfaction with the educational intervention. Questions assessing satisfaction addressed enjoyment of the interactive workshop, preference of the interactive workshop in comparison with traditional didactics, and recommendation to offer the workshop in the future. The survey also queried subjective knowledge improvement and likelihood of recommending the 20/20 SIM tool. All instruments were administered electronically via research electronic data capture. Students were informed that participation was voluntary, anonymous, and had no impact on neurology clerkship grade.

The same posttest was sent out to all participants approximately 6 months after the intervention to assess for knowledge retention. Aggregated scores from the pretests and posttests were compared, and paired *t* tests were used to analyze knowledge gain. Measure of effect size was calculated using Cohen *d*, and its prespecified interpretation was determined based on formally suggested benchmarks.^25^ Descriptive statistics were used to describe exit survey responses. For ease of interpretation, “agree” and “strongly agree” as well as “disagree” and “strongly disagree” were combined unless otherwise indicated.

A statistically significant increase in preintervention and postintervention knowledge test scores was the expectation for demonstration of improvement in understanding. Positive outcome for satisfaction was considered most of the students expressing enjoyment of the workshop, preference to traditional didactics, and recommendation to offer the session in the future.

### Standard Protocol Approvals, Registrations, and Patient Consents

This study was approved as exempt research by the ISMMS Institutional Review Board.

### Data Availability

Anonymized data not published within this article will be made available by request from any qualified investigator.

## Results and Assessment Data

From July 2020 through June 2021, we held 10 one-hour learning sessions. A total of 145 students participated, with a range of 8–18 students attending each session. In total, 125 (86%) students completed at least part of the pretest, and 102 (70%) and 88 (61%) completed at least part of the posttest and exit survey, respectively. The pretest had 5 (4%) responses and posttest 2 (2%) responses with at least 1 skipped question, respectively.

### Learner Knowledge

**Figure 2:**
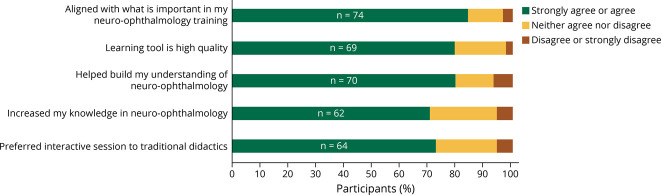
Assessment of Participant Satisfaction Exit survey results revealed that students overall thought highly of the neuro-ophthalmology learning tool and interactive workshop.

**Figure 3:**
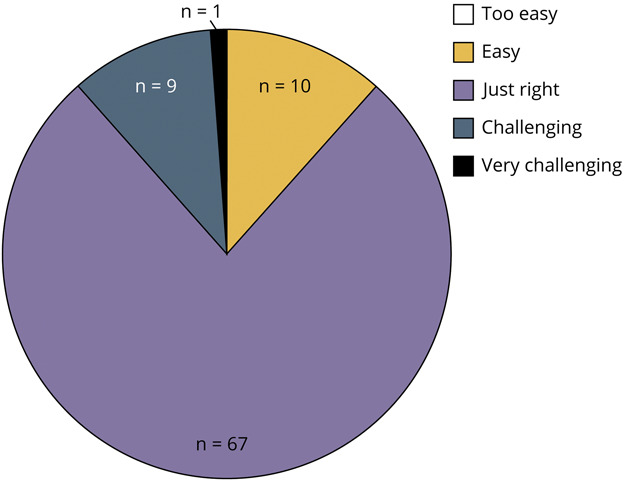
Participant Perception of 20/20 SIM Case Difficulty Most of the students (77%, n = 67) reported the difficulty of the 20/20 SIM neuro-ophthalmology cases were “just right.” Approximately 11% (n = 10) rated the cases as “challenging” (n = 9) or “very challenging” (n = 1), and the remainder thought the cases were “easy” (n = 10). None thought the tool was “too easy.”

The mean knowledge score increased from 7.5 (SD 2.2) to 8.5 (SD 1.6) of 10, *p* = 0.00014 (*d* = 0.5). Students felt that the 20/20 SIM modules aligned with what is important in their neuro-ophthalmology training (84%, n = 74), helped build their understanding of (80%, n = 70), and increased their knowledge in neuro-ophthalmology (70%, n = 62) (Figure 2). Most students felt the difficulty of the cases were “just right” (77%, n = 67), while 11% (n = 10) found them “challenging” or “very challenging” and 11% (n = 10) thought they were “easy” (Figure 3).

### Learner Satisfaction

**Figure 4:**
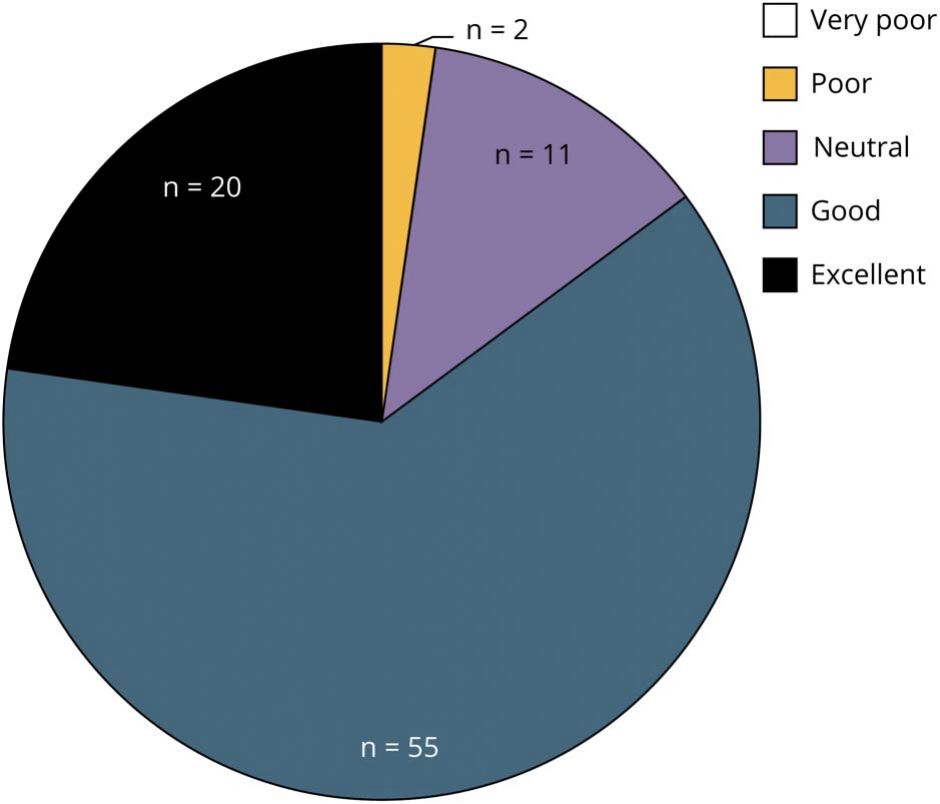
Participant Rating of the 20/20 SIM Tool Most (85%, n = 75) of the students rated the 20/20 SIM learning tool positively, with 63% (n = 55) describing it as a “good” and 23% (n = 20) as an “excellent” learning tool. Approximately 2% (n = 2) rated the tool as “poor,” and the remainder (n = 11, 13%) were neutral. None rated it as “very poor.”

Most of the students believed that the learning tool was of high quality (79%, n = 69) and wished to see a similar learning tool for other specialties (92%, n = 81). In addition, most of the (85%, n = 75) students rated the 20/20 SIM learning tool positively, with 63% (n = 55) describing it as a “good” and 23% (n = 20) as an “excellent” learning tool (Figure 4). More than half of the students indicated that they were “likely” or “very likely” to recommend this neuro-ophthalmology learning tool to others (67%, n = 67), while 5% (n = 4) were “unlikely” or “very unlikely” to recommend and the remainder were “neutral.” Most of the students expressed satisfaction with the workshop, with 64% (n = 56) expressing enjoyment and 73% (n = 64) preferring the interactive session to traditional didactics. Only 6% (n = 5) preferred traditional didactics, and the remainder of participants felt neutral between the 2. Almost all students recommend the learning sessions be offered in the future (92%, n = 81).

### Platform Utilization

Approximately one-fifth (18%, n = 16) of the students who completed the exit survey reported visiting the website in the 4 weeks between the pretest and the posttest at least once. From July 2020 through March 2022, the neuro-ophthalmology section of 2020SIM.com had 935 case views. Views of each of the 5 cases ranged from 5 to 220, and case completion rates ranged from 0.40 to 0.99 from 2020 to 2022. While a follow-up posttest was administered 6 months, the response was poor at n = 3. Therefore, we were not able to assess long-term knowledge retention.

## Discussion and Lessons Learned

We developed and studied a 1-hour virtual interactive workshop, designed to improve subjective and objective understanding of neuro-ophthalmologic manifestations of common neurologic conditions. Using this FOAMed tool during the neurology clerkship, we also evaluated participant satisfaction of the associated workshop. We used the same platform and maintained the stepwise question style as 2020SIM.com, based off of NephSIM.^17^ In the first year of this educational intervention, we demonstrated subjective and objective improvement in student understanding of the neuro-ophthalmologic manifestations of common neurologic conditions during the neurology clerkship. Student knowledge scores on the learner assessment showed a statistically significant increase by 13%, and most of the students felt the platform helped increase their knowledge of neuro-ophthalmology. In addition, almost three-quarters of students preferred the interactive session to traditional didactics, and almost all recommended the workshop be offered in the future. One-fifth of students returned to the 20/20 SIM website outside our workshop, and our web hit data suggest a large number of people visited the site outside of our workshop cohort. Our experience suggests that the integration of an interactive online learning tool, such as the 20/20 SIM platform, may be an effective method to build knowledge in topics with less curricular exposure.

Integrating neuro-ophthalmology education into the neurology clerkship can help ensure that all graduating medical students gain at least some exposure to the field despite the varied experiences students inevitably have on rotations. One medical school implemented a supplemental online neurology lecture course to help standardize didactic teaching during their 4-week rotation. Similarly to the 20/20 SIM layout, the course emphasized symptom-based learning and ended with a quiz using video clips and clinical images. Students who participated in the novel course earned higher scores on clinical quiz and shelf examination and reported higher levels of satisfaction for the neurology didactics.^26^

Other institutions have introduced online-based educational modalities in neuro-ophthalmology. At one teaching hospital, a virtual elective in neuro-ophthalmology was implemented to help combat the lack of clinical exposure during the coronavirus disease 2019 pandemic. Unlike our interactive workshop, the format simulated that of a traditional elective including traditional lectures (i.e., morning report, grand rounds), clinical and research experiences, and examination that were all converted to a virtual setting over 4 weeks.^27^ While this type of comprehensive alternative to in-person teaching is a valuable learning resource, we sought to introduce a FOAMed tool in a case-based format that is easy to implement into an already existing clerkship infrastructure or outside of formal curricular time. The case-based workshop with our supplemental teaching guide (eAppendix 1, links.lww.com/NXG/A582) allows educators even without neurology or ophthalmology expertise to teach high-yield neuro-ophthalmology.

Approximately three-fourths (73%, n = 64) of participants preferred our interactive case-based discussion to traditional didactics. Very few preferred traditional didactics (6%, n = 5), and approximately one-fifth (22%, n = 19) of students in our study were neutral between the 2 lecture types, which can support hybrid learning environments with multiple teaching styles to accommodate a variety of educational needs. The interdisciplinary content covered as well as the collaboration between specialists from both fields in the case development uniquely provided learners with dual perspectives on approaching such cases, which is indicative of future collaboration in clinical practice.

The interactive format including cases, questions with explanations, and embedded hyperlinks for additional learning beyond the 20/20 SIM platform allows for broad dissemination of knowledge because the tool can be accessed beyond our institution and medical student cohort by interested learners worldwide. Although we introduced this platform during our neurology clerkship interactive workshop partly to assess its effectiveness and student satisfaction, no formal curricular time is necessarily required to use this learning tool. The cases were developed with the intention that trainees can independently on their own time navigate the cases and mimic real-life practice. If incorporating this or a similar tool into a formal trainee learning environment, it requires few resources and is easy to implement. Our supplemental teaching guide (eAppendix 1, links.lww.com/NXG/A582) provides the logistics, learning objectives, detailed instructions, and key learning points to highlight during the interactive session.

Our web hit data suggest a broad reach of 2020SIM.com, given the high number of case views well beyond the number of participants in our workshops. Our data suggest that almost one-fifth of students visited the website outside our workshops, which was more than our study team had anticipated, given the clinical and educational requirements of the clerkship and abundance of study resources clerkship students are accustomed to using in preparation for examinations. We are unfortunately unable to determine the quantity of page visits that were related to our workshops.

Our findings of knowledge gains and overall student satisfaction suggest that similar tools may be effective in increasing exposure to and knowledge of other disciplines. While our data do not establish which factors contribute most to the observed increase in student knowledge, we suspect the greatest contributions are from the 1-hour workshop because most of the students (80%) did not return to the website outside the workshop. The increase in knowledge may also be due to on-site clerkship learning and alternative study resources (e.g., question banks); however, this is likely limited, given the minimal neuro-ophthalmology content available then for medical trainees. Our tool may help with achieving basic competency in the evaluation of common neuro-ophthalmic presentation among all medical students and other learners who engage with the learning tool. While we did not measure increased interest in neuro-ophthalmology, neurology, or ophthalmology after the session, we are hopeful our intervention may ultimately contribute to increased recruitment in such fields.

We acknowledge several limitations. First, the single-center nature of our study may limit external validity; however, it included a relatively large sample size of more than 100 students across 10 months. Varying cases were discussed during each workshop; however, there was considerable overlap, given that there were only 5 possible cases and each session discussed 2–3 cases. Incomplete responses may have affected our results because skipping a question or answering it incorrectly were both scored as zero points. However, very few questions were skipped on the knowledge questionnaire. In addition, given the weeks between the pretest and posttest during the neurology clerkship and multiple learning opportunities throughout the clerkship, it is not possible to determine what proportion of knowledge acquisition is attributed to our intervention. However, we know that exposure to these topics is limited in the traditional education setting, and most of the students reported that our tool helped build their understanding of and increased knowledge in neuro-ophthalmology concepts. Last, our questions were original and not from previously validated sources. However, each was based on a review of available literature and question banks as well as peer reviewed by our neurology (L.K.S., M.F.) and ophthalmology (N.C.) faculty.

We explored learner subjective and objective knowledge acquisition and satisfaction of the educational tool in an interactive workshop, which can represent another use case for e-learning and CBL. Our study revealed that clerkship medical students learned from the 1-hour virtual interactive workshop, preferred CBL to traditional didactics, and recommended a similar online platform in other specialties. Our experience suggests that the integration of interactive online learning tools, such as the SIM platform, into curricular workshops offers a favorable and effective strategy to increase exposure to topics with less curricular time. In the future, such tools can be developed in a variety of subspecialties and incorporated into graduate medical education as well.

## Appendix Authors

**Table:**

Name	Location	Contribution
Emma M. Loebel, MD	Department of Neurology, Icahn School of Medicine at Mount Sinai, New York City	Analyzed and interpreted the data, drafted and revised article for intellectual content
Laura K. Stein, MD, MPH	Department of Neurology, Icahn School of Medicine at Mount Sinai, New York City	Designed and conceptualized the study, revised article for intellectual content
Michael Fara, MD, PhD	Department of Neurology, Icahn School of Medicine at Mount Sinai, New York City	Revised article for intellectual content, major role in acquisition of data
Samira Farouk, MD	Department of Nephrology, Icahn School of Medicine at Mount Sinai, New York City	Revised article for intellectual content
Nisha Chadha, MD	Department of Ophthalmology, Icahn School of Medicine at Mount Sinai, New York City	Designed and conceptualized the study, revised article for intellectual content

## Glossary

CBL: case-based learning
FOAMed: free open-access medical education
HPI: history of present illness
ISMMS: Icahn School of Medicine at Mount Sinai
